# Electrical characteristic fluctuation of 16-nm-gate trapezoidal bulk FinFET devices with fixed top-fin width induced by random discrete dopants

**DOI:** 10.1186/s11671-015-0739-0

**Published:** 2015-03-11

**Authors:** Wen-Tsung Huang, Yiming Li

**Affiliations:** Parallel and Scientific Computing Laboratory, National Chiao Tung University, 1001 Ta-Hsueh Road, Hsinchu, 300 Taiwan; Institute of Communications Engineering, National Chiao Tung University, 1001 Ta-Hsueh Road, Hsinchu, 300 Taiwan; Department of Electrical and Computer Engineering, National Chiao Tung University, 1001 Ta-Hsueh Road, Hsinchu, 300 Taiwan

**Keywords:** Random dopant fluctuation, Characteristic fluctuation, Short-channel effect, Bulk FinFET, Channel fin angle, Trapezoidal, Ideal channel fin, Nonideal channel fin, Top-fin width

## Abstract

In this work, we use an experimentally calibrated 3D quantum mechanically corrected device simulation to study the random dopant fluctuation (RDF) on DC characteristics of 16-nm-gate trapezoidal bulk fin-type field effect transistor (FinFET) devices. The fixed top-fin width, which is consistent with the realistic process by lithography, of trapezoidal bulk FinFET devices is considered in this study. For RDF on trapezoidal bulk FinFETs under the fixed top-fin width, we explore the impact of geometry and RDF on the on-/off-state current and the threshold voltage (*V*_th_) fluctuation with respect to different channel fin angles. For the same channel doping concentration, compared with an ideal FinFET (i.e., device with a right angle of channel fin), the off-state current is large in trapezoidal bulk FinFETs with a small fin angle. Furthermore, the short-channel effect and *V*_th_ variation degrade as the fin angle is getting smaller. The magnitude of the normalized *σV*_th_ increases 7% when the fin angle decreases from 90° to 70°.

## Background

Scaling down the CMOS technology node beyond the sub-20 nm causes the transistor to go through a transition from planar to multi-gate FETs such as bulk fin-type field effect transistors (FinFETs) because of the requirement of better gate control and suppression on short-channel effects (SCEs) [1-3]. In addition to the improvement on DC characteristics of individual device, however, continuously scaling not only overcomes challenges on fabrication but also suppresses systematic variation and random effects [4,5]. In practical fabrication, it is difficult to obtain uniform thickness along the height of the fin channel due to limitations in process technology [6]. The actual fins channel may be fabricated as trapezoidal shape and degrade the device performance by significant SCEs. For variability issues, there are many serious fluctuation sources such as random dopant fluctuation (RDF) [7], work-function fluctuation [8], interface trap fluctuation [9], and the line edge roughness [10]. For low-standby-power device technologies and applications, channel doping is still needed to adjust the threshold voltage (*V*_th_) and RDF has been shown as the major source of variations for high-Κ metal gate (HKMG) bulk FinFET devices [11] among various fluctuation factors. Recent researches on RDF and fin-shape effects were reported for FinFET devices; however, the studies on FinFET’s RDF were only considered for devices with a rectangular-shape fin channel [12]. However, the channel fin is not always with an ideal shape owing to process challenges. To the best of our knowledge, a research which simultaneously considers the aforementioned issues has not been well investigated yet.

In this study, we explore the RDF on DC characteristics of 16-nm-gate trapezoidal bulk FinFETs with the fixed top-fin width (*W*_top_) condition. We further intensively analyze the on-/off-state current characteristic and *V*_th_’s fluctuation of the 16-nm-gate trapezoidal HKMG bulk FinFET. The article is organized as follows. The ‘Methods’ section introduces the simulation technique for studying the RDF on trapezoidal bulk FinFET devices with different fin angles and the same *W*_top_. The ‘Results and discussion’ section focuses mainly on the analysis and discussion of characteristic fluctuation from RDF of 16-nm-gate trapezoidal HKMG bulk FinFET devices. Finally, we draw conclusions and suggest future work.

## Methods

### The device configuration and simulation technique

**Figure 1:**
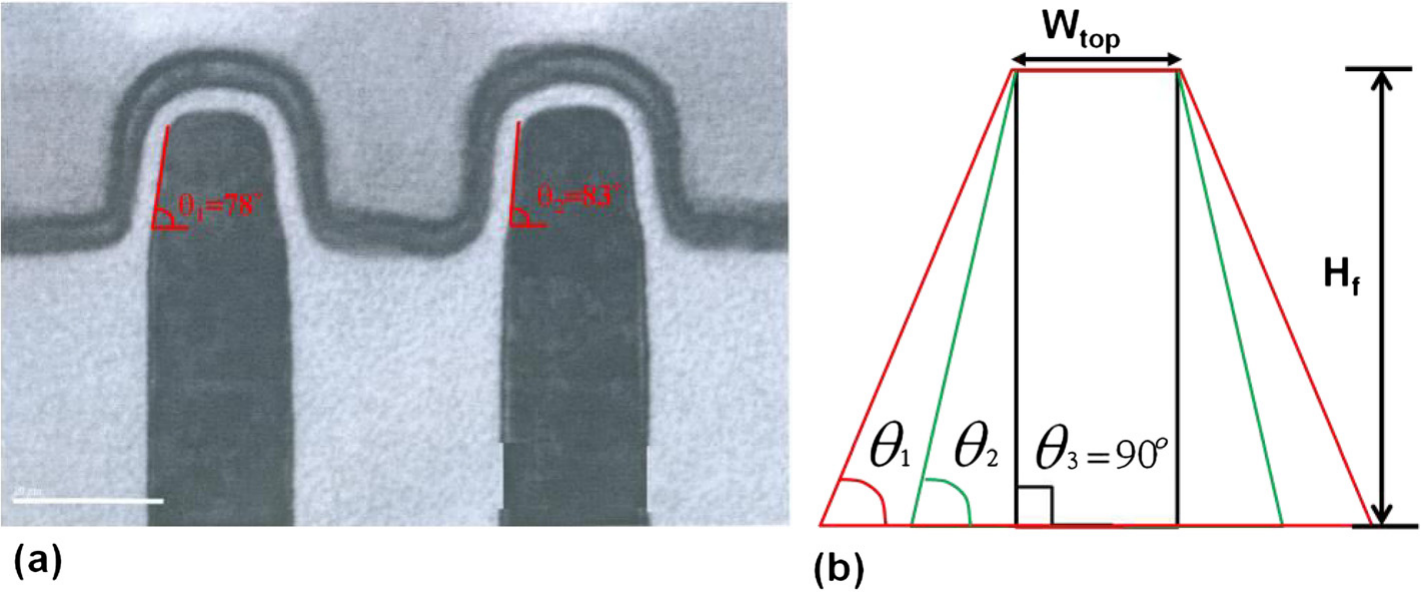
**TEM and schematic cross-section views of channel fin. (a)** A TEM view of realistic shape of fabricated channel fin to show the fin angle variation. **(b)** The schematic cross-section view of channel fin with respect to different fin angles for devices with a fixed-top-fin width (*W*_top_), in this study.

Figure 1a shows various TEM views of realistic shape of fabricated two-channel fin in 16-nm technological node. Due to the process capabilities, the fin angles *θ*_1_ and *θ*_2_, as shown in these two images, may vary with the lithography and etching, etc. Therefore, devices with different trapezoidal shapes are fabricated. In this study, we assume the top-fin width is fixed at 8 nm for the 16-nm-gate HKMG bulk FinFET devices. Figure 1b shows the schematic cross-section view of channel fin for the identical-*W*_top_ setting. The fin angle (*θ*) is thus defined as the angle between the bottom line and sidewall and ranges from 70° to 90°. Besides the fixed *W*_top_, the channel doping concentration, which means the needed number of impurity in the channel region increases when the fin angle is getting smaller, is set the same for all simulation cases.

**Figure 2:**
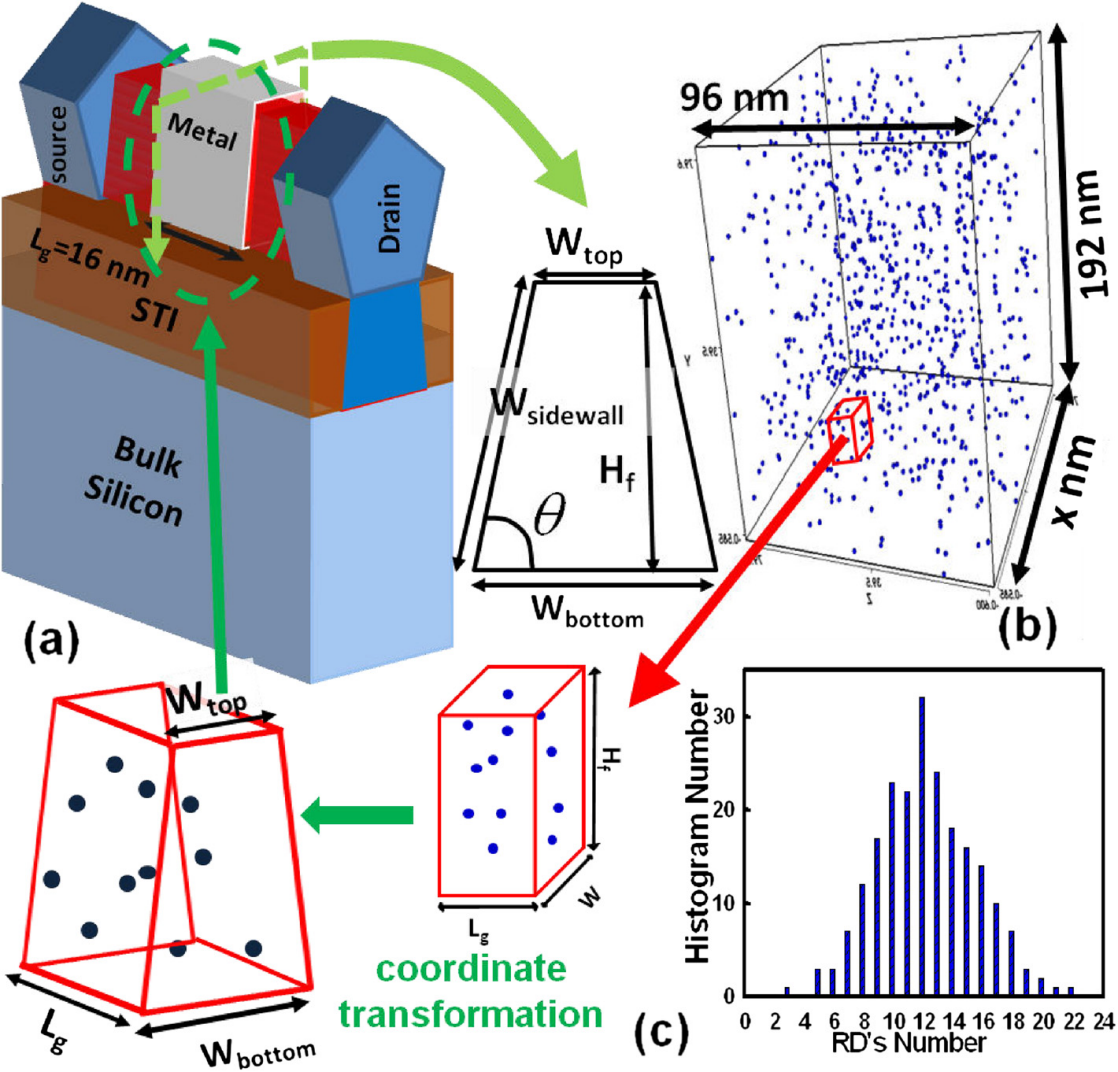
**The large-scale statistical device simulation method of RDF on the device’s channel region. (a)** The schematic structure of the 16-nm-gate trapezoidal bulk FinFET device and the cross-section view of fin channel. **(b)** Discrete dopants are randomly distributed in a large cube with the average concentration of 1.5 × 10^18^ cm^−3^. The number of RDs is dependent on the magnitude of the fin angle. **(c)** The right plot is the bar chart of distribution of number of dopants of each fin-angle bulk FinFET. The left plot is the procedure of generating fluctuated cases. The cube is partitioned into many subcubes. The coordinate transform is performed on those subcubes to make them become trapezoidal-shaped channels and map them into device channel regions.

Figure 2a shows the simulated bulk FinFET’s structure and the cross-section of fin channel. Table 1 lists the simulation settings and the achieved DC-characteristic parameters of each trapezoidal FinFET. The *W*_top_, bottom-fin width (*W*_bottom_), and the total fin width (*W*_total_), which is defined as 2 × *W*_sidewall_ + *W*_top_, are also listed in Table 1. The value of *W*_total_ is getting larger when the fin angle is getting smaller, and it is related to on-state current. The effective work function ranges from 4.4 to 4.5 eV, which is used to adjust the value of *V*_th_. The constant current method at 0.1 μA × *W*_total_/*L*_g_ is used to extract the magnitude of *V*_th_, and the absolute value of the nominal *V*_th_ of each different-fin-angle trapezoidal FinFET is 250 mV. For the comparison of RDF on trapezoidal bulk FinFETs, the simulation is based on the same *V*_th_, where the adopted device parameters are listed in Table 1. Figure 2b,c shows the large-scale statistical simulation method of RDF on the channel region. For RDF simulation, many discrete dopants dependent on the geometry are randomly generated in a large cube, where the dopant concentration in the large cuboid is equivalent to a channel doping concentration of 1.5 × 10^18^ cm^−3^. Then, the large cube is partitioned into many subcubes, where the distribution of RDs’ number follows Gaussian distribution, as shown in the right plot of Figure 2c. Then, a transformation of coordinate is performed on those subcubes to make them become trapezoidal-shaped channels and map them into 3D device channel regions. Notably, each coordinate of RDs is also transformed so that they appear in the trapezoidal-shaped channel exactly. To investigate the devices’ characteristics, a set of 3D drift-diffusion equations coupled with the density gradient equation for quantum correction is solved [13,14]. The mobility model used in the device simulation mainly follows our earlier work [15] which involves surface roughness, high-field saturation, and impurity scattering. Notably, the mobility model activated in our device simulation considers the influence of surface orientations on the on-state current by the term of effective electric field for every fin angle [16]. The mobility model is quantified with our recent device measurements for the best accuracy of simulation, and the characteristic fluctuation has been validated with the experimentally measured DC base band data from 15/20 CMOS devices [15]. For each statistical device simulation, 216 RD-fluctuated FinFET devices are randomly generated for every fin angle to estimate the magnitude of the RDF-induced characteristic fluctuation.

**Table 1:** **The simulation settings of nominal bulk FinFET devices**

**Fin angle** ***θ*** **(**°**)**	**90**	**85**	**80**	**75**	**70**
Top-fin width, *W*_top_ (nm)	8	8	8	8	8
Bottom-fin width, *W*_bottom_ (nm)	8	13.6	19.3	25.1	31.3
Total fin width, *W*_total_ (nm)	72	72.24	72.99	74.26	76.1
Fin height, *H*_f_ (nm)	32				
Effective oxide thickness, EOT (nm)	1				
Gate length, *L*_g_ (nm)	16				
Source/drain doping (cm^−^3)	1.0E20				
Punch-through stopper (cm^−^3)	1.5E19				
Channel doping (cm^−^3)	1.5E18				
Drain voltage, *V*_DD_ (V)	0.8				

The *V*_th_ are calibrated to 250 mV.

## Results and discussion

**Figure 3:**
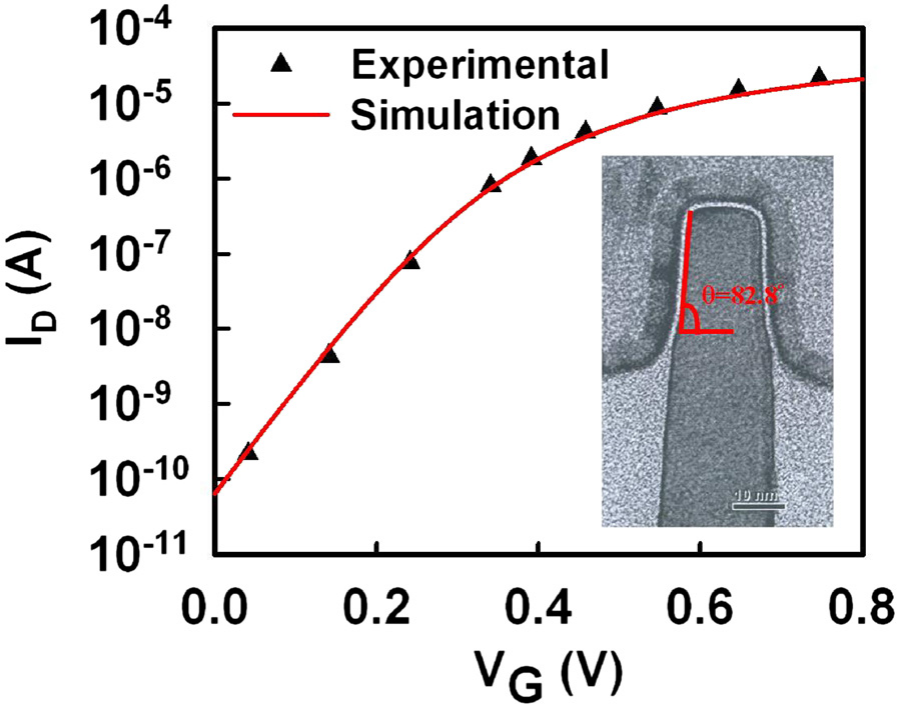
**Plot of calibrated** ***I***_**D**_ **-** ***V***_**G**_ **curves.** The red solid line is the *I*_D_-*V*_G_ of simulation result, and the triangular symbols are experimental data. The inset shows the TEM cross-section view of the fabricated and measured device.

The inset of Figure 3 shows a TEM cross-section view of fabricated 16 nm n-type bulk FinFET device (gate length *L*_g_ = 16 nm) with amorphous-based TiN/HfSiON gate stacks with an EOT of 1.0 nm. The channel fin width is 16 nm, and the fin height is 32 nm. To ensure the best accuracy of device simulation, the *I*_D_-*V*_G_ curve of the FinFET at *V*_D_ = 0.8 V is experimentally calibrated with measured data (symbols), as shown in Figure 3, where the extracted physical and process parameters are used for the following study.

**Figure 4:**
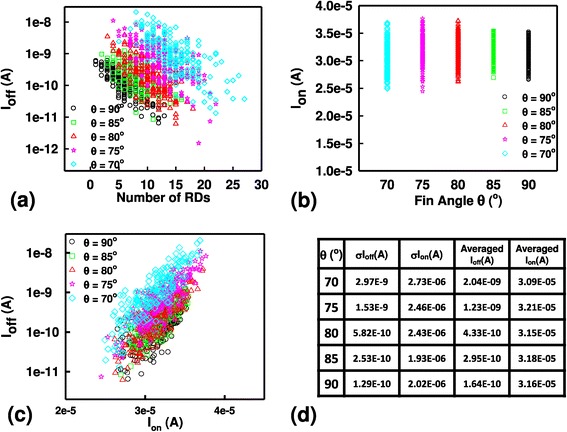
**Plots of** ***I***_**off**_ **versus** ***I***_**on**_ **and values of** ***σI***_**off**_ **,** ***σI***_**on**_ **, averaged** ***I***_**off**_ **, and averaged** ***I***_**on**_ **. (a)** The *I*off versus RDs’ number plot. **(b)** The *I*_on_ versus RDs’ number plot. **(c)** The on/off current characteristic plot. **(d)** The value of *σI*_off_, *σI*_on_, averaged *I*_off_, and averaged *I*_on_ of trapezoidal FinFET device with different fin angles.

Figure 4 shows the on-/off-state current characteristics of the trapezoidal bulk FinFETs with the fixed *W*_top_. The plot of *I*_off_ versus RDs’ number is shown in Figure 4a. The magnitude of *I*_off_ of rectangle-shaped bulk FinFETs is small, and 70° bulk FinFET has large *I*_off_ . In addition, the distribution of *I*_off_ is getting more dispersive when the fin angle is getting smaller. Under the same *W*_top_, the phenomena are as a result of the weak lateral gate control of large bottom-fin width (i.e., small fin angle). The plot of *I*_on_ versus RDs’ number is shown in Figure 4b. Though the *W*_total_ of 70° bulk FinFET is large, the on-state should be the largest of all. However, in rectangle-shaped bulk FinFETs, the fin width is narrow enough to induce strong volume inversion, where the electron mobility is enhanced due to less surface roughness. Therefore, the on-state current is comparable in the rectangle-shape bulk FinFET with the 70° bulk FinFET. Figure 4c shows the plot of *I*_off_ versus *I*_on_. The FinFET with large fin angle has better on-/off-state current ratio and wider distribution of on-/off-state current than that of the FinFET with relatively smaller fin angles.

**Figure 5:**
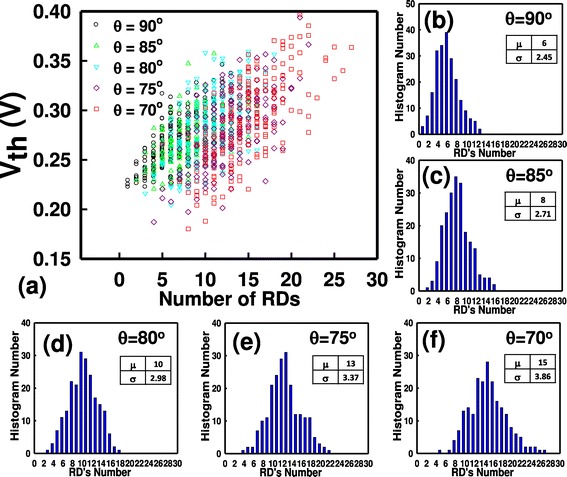
**Plot of** ***V***_**th**_ **versus the number of RDs and the RDs’ number distribution. (a)** The *V*th versus the number of RDs. **(b) to (f)** RDs’ number distribution for every fin angle, where the *μ* is the mean and *σ* is the fluctuation.

Figure 5 shows the plot of the *V*_th_ versus the number of RDs and the RDs’ number distribution for every fin angle. We note that each RD has the same size and concentration; therefore, the bulk FinFET with a small fin angle needs more amount of RDs’ number to achieve the same channel concentration due to large channel volume. As shown in Figure 5a, the trend of *V*_th_ fluctuation is dominated by RDs’ number distributions, as shown in Figure 5b,c,d,e,f. The bulk FinFET whose fin angle is right angle has the smallest *σV*_th_. It has been known that the calculation of *σV*_th_ follows the equation [17]:

1 $$ \sigma V\mathrm{t}\mathrm{h},\mathrm{R}\mathrm{D}\mathrm{F}=\frac{A_{\mathrm{VT}}}{\sqrt{W\times {L}_{\mathrm{g}}}}, $$

where *A*_VT_ is the factor which is governed by manufacturing process and semiconductor material, *W* stands for the device’s width, and *L*_g_ is the gate length. The denominator is dependent on the dimension and structure of devices. If we simply consider *W* as the effective channel width, which is defined by 2 × *H*_f_ + *W*_top_ for the rectangular-shape FinFET (or 2 × *W*_sidewall_ + *W*_top_ for various trapezoidal FinFETs), it cannot be used to explain our results of Figure 5a. It is because the estimation of Equation 1 is originally derived from planar MOSFETs, based on an assumption of the ability on inducing inversion charge is the same for whole gate region.

**Figure 6:**
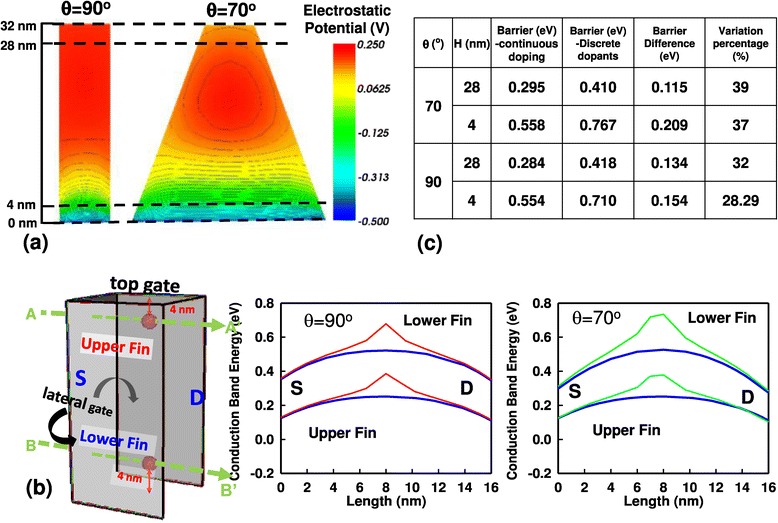
**Off-state potential distribution of FinFET device and influence of one dopant on conduction band energy. (a)** The off-state potential distribution of FinFET device with the right angle and 70° fin angle. **(b)** The influence of one dopant on upper-fin and lower-fin conduction band energy of FinFET device with the right angle and 70° fin angle. **(c)** The related value in **(b)**, which can indicate the influence of dopant is dependent on the position and the fin angle and the ability of inducing inversion charge is not the same everywhere of gate.

For FinFET devices, the ability of 3D vertical channel structure on inducing inversion charge is not the same due to the different coupling strengths of the top gate and the lateral gates. This phenomena could also be observed by examining the off-state (*V*_G_ = 0 V) potential distribution, as shown in Figure 6a, where the high potential region is easy to induce charge and the low potential region in the lower fin would block off most transport electrons. As shown in plots of Figure 6b, if we slice the channel fin of bulk FinFET device (the left plot is for an ideal structure) along the lines in green, the conduction band energies of continuous-doping device and discrete-dopant device (we assume there is 1 RD on upper/lower fin in this case, respectively) at upper/lower fin are obtained, respectively. The same way is also applied on the 70° trapezoidal FinFET. The right two plots of Figure 6b indicate the off-state potential energies, and their fluctuated barriers are rather different not only for RDs appearing in different fin regions but also for devices with different fin angles.

**Figure 7:**
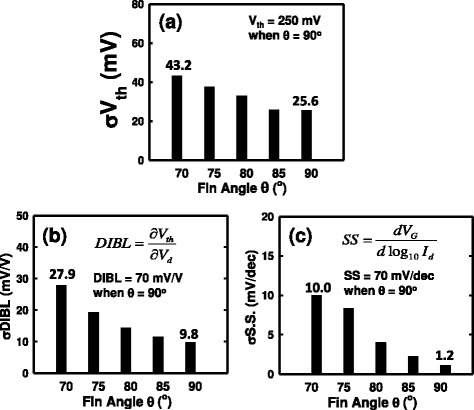
**RDF stimulation result and magnitudes of** ***σV***_**th**_ **,** ***σ*** **DIBL, and** ***σ*** **SS. (a)** The RDF simulation result of bulk FinFET devices with respect to different fin angles. The magnitude of *σV*_th_ is getting larger when the fin angle is getting smaller. **(b)** The magnitude of *σ*DIBL versus the fin angle. The magnitude of *σ*DIBL increases when the fin angle decreases owing to the wider bottom-fin width. **(c)** The magnitude of *σ*SS versus the fin angle.

Consequently, the influence of RDs on device’s subthreshold region is strongly dependent on the RDs’ position, the shape of channel, and the fin angle. Thus, the fin-angle-dependent ability on inducing inversion charge is indeed not the same for the entire gate region. Therefore, the argument of *W*_top_ is not suitable to be regarded as the *W* in Equation 1. The effective *W* would be decreased with the fin angle getting smaller, and *σV*_th_ will be increased. The dopant variation induced *V*_th_ fluctuation is significant when the fin angle is small due to the less gate control. Therefore, based on the simulation results of Figures 4 and 5, in particular, from the analysis of RD’s position effect, we further propose an analytical expression to phenomenologically correct Equation 1:

2 $$ \sigma V\mathrm{t}\mathrm{h}=\frac{A_{\mathrm{VT}}^{\hbox{'}}\times {\left[\left(a\times {e}^{-b\times \theta}\right)\times {N}_{\mathrm{ch}}\right]}^{0.25}}{{}^{{\left({W}_{\mathrm{total}}\times {L}_g\right)}^{0.25}}}, $$

where the fitting coefficients *a* = 5.5 × 10^5^ and *b* = 0.12. The ${A}_{\mathrm{VT}}^{\hbox{'}}$ is a technologically dependent parameter except the channel dopant concentration and *N*_ch_ is the channel doping concentration and *θ* is the fin angle which ranges from 70° to 90°. Notably, the denominator is dependent on the dimension and structure of devices and the factor 0.25 is mainly determined from the device structure which is different from the value of 0.5 used for planar MOSFET devices [18]. Two significant factors could be distinguished from this formula: one is the so-called structural effect as a result of the *W*_total_ under the fixed gate length, and the other is related to the RDs’ position effect. The relationship between the effective channel dopants and the magnitude of the fin angle follows the trend of exponential decay. The influence of the fin angle dependent effective channel dopants dominates *V*_th_ fluctuation as the result of the small variation on the magnitude of *W*_total_ from variation of the fin angles. Not shown here, our model prediction is within 3% of accuracy, compared with the results in Figure 7a.

As shown in Figure 7, we estimate the fluctuations of the threshold voltage, the drain-induced barrier lowering (DIBL), and the subthreshold swing (SS) with respect to the fin angle. Consequently, as shown in Figure 7a, the *V*_th_ fluctuation decreases when the fin angle increases for the trapezoidal bulk FinFET devices under the constant top-fin width. There is more than 7% increase on the *V*_th_ fluctuation when the fin angle varies from 90° to 70° $\left(\left(\frac{43.2-25.6}{250}\right)\times 100\%\approx 7\%\right)$, where the denominator is the nominal *V*_th_ = 250 mV when *θ* = 90°. Figure 7b,c shows the plots of the fluctuations of DIBL (*σ*DIBL) and SS (*σ*SS) versus the fin angle, respectively. Both DIBL and SS fluctuations are getting significant when the fin angle is getting smaller. The relationship between DIBL as well as SS fluctuation and the fin angle corresponds with the *V*_th_ analysis. The bulk FinFETs with a small fin angle suffers serious *σ*DIBL (about 25.9% increases) due to large fin width. The increase of *σ*SS is about 12.6% when the fin angle varies from 90° to 70°.

## Conclusions

RDF on trapezoidal bulk FinFET devices with the fixed *W*_top_ and different fin angles is studied by experimentally validated 3D device simulation. The bulk FinFET devices with large fin angle have small off-state current due to the strongest gate control. For the tested channel fin width of 8 nm, the on-state current of the 16-nm-gate HKMG bulk FinFET device is almost the same for all trapezoidal-shaped channels. Furthermore, *V*_th_’s fluctuation is affected by RDs’ number distribution under the same channel doping concentration and the right-angle bulk FinFET device has the smallest *V*_th_’s fluctuation among all devices with nonideal channel fins. We note that Equation 1 should be subject to further investigation for the calculation of *σV*_th_ of bulk FinFET devices.

Definitely, an assumption that fixed the bottom-fin width and let the top-fin width be varied with the fin angle could be an interesting issue to be investigated in a future work. In addition, for bulk FinFET devices, a punch-through stopper is adopted for reducing the subthreshold leakage. The punch-through stopper may result in another RDF source owing to high substrate doping near the bottom channel. RDF simulation with including the impact of punch-through stopper could be subjected to further investigation, and it definitely will input more accurate estimation on the characteristic fluctuation.

## Abbreviations

FinFET: fin-type field effect transistor
RDF: random dopant fluctuation
RD: random dopant
SCE: short-channel effect
*V*_th_: threshold voltage
*W*_top_: top-fin width
*W*_bottom_: bottom-fin width
